# Kui Jie Tong Ameliorates Ulcerative Colitis by Regulating Gut Microbiota and NLRP3/Caspase-1 Classical Pyroptosis Signaling Pathway

**DOI:** 10.1155/2022/2782112

**Published:** 2022-07-04

**Authors:** Shigui Xue, Yan Xue, Danbo Dou, Huan Wu, Ping Zhang, Yang Gao, Yini Tang, Zehua Xia, Sen Yang, Sizhen Gu

**Affiliations:** ^1^Department of Digestive Endoscopy Center, Shuguang Hospital Affiliated to Shanghai University of Traditional Chinese Medicine, Shanghai 201203, China; ^2^Shanghai Yangzhi Rehabilitation Hospital (Shanghai Sunshine Rehabilitation Centre), Shanghai 201619, China; ^3^Department of Traditional Chinese Medicine, Shuguang Hospital Affiliated to Shanghai University of Traditional Chinese Medicine, Shanghai 200032, China; ^4^Department of Anorectal, Shuguang Hospital Affiliated to Shanghai University of Traditional Chinese Medicine, Shanghai 201203, China; ^5^Department of Clinical Lab, Shuguang Hospital Affiliated to Shanghai University of Traditional Chinese Medicine, Shanghai 201203, China

## Abstract

Ulcerative colitis (UC) is one of the most refractory digestive diseases in the world. Kui jie tong (KJT) is an effective traditional Chinese medicine used clinically to treat UC. This study observed the regulatory effects of KJT on NIMA-related kinase 7- (NEK7-) activated nod-like receptor protein-3 (NLRP3)/caspase-1 classical pyroptosis pathway and intestinal flora in UC model rats. KJT components were analyzed using an ultraperformance liquid chromatography-tandem mass spectrometer (UPLC-MS/MS). A UC Sprague Dawley (SD) rat model was established using sodium dextran sulfate (DSS). Rats were randomly divided into four groups: control group (CG), UC model group (UG), KJT group (KG), and sulfasalazine (SASP) group (SG). After seven days of intervention, each group's body weight, disease activity index (DAI) scores, and colon length were recorded. Intestinal mucosal injury to each group was observed using hematoxylin-eosin staining. Additionally, we investigated the expression levels of NEK7, NLRP3, ASC, caspase-1, and GSDMD in intestinal mucosa, as well as serum interleukin- (IL-) 1*β*, IL-18, and IL-33 proinflammatory factors. Intestinal microflora was analyzed using 16s rRNA sequencing. KJT controlled weight loss; decreased DAI scores; restored colon length; improved pathological injury in the colon; inhibited NEK7, NLRP3, ASC, caspase-1, cleaved-caspase-1, GSDMD, and GSDMD-N expression; and decreased IL-1*β*, IL-18, and IL-33 contents in UG rats' serum and colon tissue (P <0.001 or P <0.05). KJT also increased Ruminococcaceae, unclassified_f_Ruminococcaceae, and unclassified_g_Ruminococcus_1 levels and decreased Erysipelotrichia, Erysipelotrichales, Erysipelotrichaceae, Turicibacter, and uncultured_bacterium_g_Turicibacter levels. KJT alleviated UC immune-inflammatory responses to NLRP3/caspase-1 by inhibiting the NEK-7-activated classic pyroptosis pathway and improving intestinal microflora.

## 1. Introduction

Ulcerative colitis (UC) is a chronic, persistent, nonspecific immune bowel disease. The condition is painful and seriously complicates patients' lives, with long-term uncontrolled UC potentially leading to colorectal cancer. World Health Organization currently lists UC as one of the most refractory digestive diseases. UC incidence has recently been steadily rising in China [1].

Currently, it is accepted that UC-mediated intestinal immune-inflammatory damage is strongly associated with flora imbalance and abnormal activation of innate immunity processes. Several studies [2] have confirmed a serious intestinal flora imbalance in UC patients and associated animal models, including decreased flora diversity and abnormal microflora structural composition [3], e.g., decreased Bifidobacterium, Lactobacillus, Rosella, and Prosnitzia and increased pathogens, i.e., Proteus, Enterococcus, and Prevoteus [4]. When there is flora imbalance, intestinal pathogen-related molecular models (PAMPs) are increased, including lipopolysaccharide (LPS), phosphoteichoic acid (LTA), peptidoglycan (PGN), mannose, and bacterial DNA. These PAMPs are recognized using pattern recognition receptors (PRRs) in the intestinal mucosa, followed by a series of proinflammatory mechanisms, which are considered important to cause intestinal mucosa immune-inflammatory injury in UC.

PRRs primarily include toll-like receptors (TLRs) and nod-like receptors (NLRs). As a vital PRR, nod-like receptor protein-3 (NLRP3) inflammatory bodies are widely distributed in intestinal mucosal epithelial cells, and inflammatory signal bodies are found in submucosal macrophages, dendritic cells, and neutrophils [5, 6]. These bodies are composed of NLRP3 [7], apoptosis-associated speck-like protein containing a CARD (ASC), and cysteinyl aspartate specific proteinase-1 (caspase-1). NLRP3 inflammatory bodies mediate immune inflammation in two stages: priming and activation: first, TLRs and other signals activate nuclear factor-*κ*B (NF-*κ*B) transcription to produce inactive pro-NLRP3 precursors (pro-NLRP3), interleukin-1*β* precursors (pro-IL-1*β*), interleukin-18 precursors (pro-IL-18), and other inflammatory factors [8]. Then, under the action of the second messenger such as ATP and microorganisms, pro-NLRP3 recruits ASC and caspase-1 to assemble an NLRP3/ASC/caspase-1 protein complex, i.e., NLRP3 inflammatory bodies, which are then activated by potassium ion efflux, reactive oxygen species, and lysosome release proteases [9–11]. The effector protein caspase-1 not only cleaves the proinflammatory cytokines IL-1*β*, IL-18, and IL-33 but also the key protein, Gasdermin-D (GSDMD) [12]. GSDMD polymerizes to lipid membranes to form regular pores, mediating the secretion and release of large numbers of mature inflammatory cytokines, followed by osmotic swelling and pyroptosis. This caspase-1/GSDMD-dependent programmed inflammatory necrosis pathway is termed the classical pyroptosis pathway [13]. NIMA-related protein kinase 7 (NEK7) [14, 15] is a key protein involved in forming and activating inflammatory bodies in NLRP3 [16, 17]. As these bodies form the core of a series of inflammatory responses, their activation and regulation in disease warrants investigation [18–21].

Recent studies have demonstrated that traditional Chinese medicine used for UC exerts considerable therapeutic advantages in terms of relieving patient conditions, improving quality of life, and improving toxic side-effects [22–24].

Kui jie tong (KJT) is an effective herbal prescription for the treatment of ulcerative colitis, which is composed of *Verbenae Herb*, *Euphorbiae Humifusae Herba*, *Arecae Semen*, *Angelicae Sinensis Radix*, and *Aurantii Fructus Immaturus* [25]. We have completed a randomized controlled clinical trial involving 60 cases of KJT prescription for mild to moderately active UC in the previous period. The study's findings indicated that following KJT treatment, abdominal pain, diarrhea, and the number of purulent and bloody stools were significantly reduced, intestinal inflammatory damage was inhibited, and c-reactive protein (CRP), sedimentation rate (ESR), and tumor necrosis factor-alpha (TNF-*α*) were decreased. All these findings indicate that KJT has a good effect on relieving clinical symptoms, controlling inflammatory activity of UC, and improving colon pathological injury [25, 26].

Under the premise that preliminary clinical trials were effective, we performed a KJT intervention study in a 5% sodium dextran sulfate- (DSS-) induced UC rat model and confirmed that (1) KJT significantly decreased disease activity index (DAI) scores in UC rats; (2) KJT significantly improved intestinal pathological injury, reduced inflammatory cell infiltration, facilitated orderly arrangement of mucosal epithelium, regularized intestinal glands, and improved goblet cell abundance; and (3) KJT inhibited interleukin- (IL-) 1*β* and tumor necrosis factor (TNF-*α*) proinflammatory factor release [27]. In another study, KJT significantly decreased p-I*κ*B*α*/I*κ*B*α*, p-NF-*κ*B p65/NF-*κ*B p65 protein expression proportion in UC rats [28]. The results of these preliminary animal experiments suggest that KJT can ameliorate the immuno-inflammatory lesions of UC by inhibiting NF-*κ*B activation, which is the initiation signal for NLRP3. As a result, we explored the mechanism of KJT in the treatment of UC from the aspects of NLRP3 inflammatory body, as well as its activator nek7 and intestinal flora.

## 2. Materials and Methods

### 2.1. Drug Preparation and Ultraperformance Liquid Chromatography-Tandem Mass Spectrometer (UPLC-MS/MS) Analysis of KJT

All drugs were provided by Shuguang Hospital affiliated with Shanghai University of Traditional Chinese Medicine (prescription number: 2020193685). KJT is composed of *Verbenae Herb* (15 g), *Euphorbiae Humifusae Herba* (15 g), *Arecae Semen* (15 g), *Aurantii Fructus Immaturus* (15 g), and *Angelicae Sinensis Radix* (15 g). Before boiling for 1 h, distilled water (750 mL and 450 mL) was added twice. The filtrate was concentrated to 25 mL after being mixed twice and filtered. Finally, a KJT crude extract was prepared at a concentration of 3 g/mL. After high-pressure sterilization, the extract was stored at 4°C. Water extraction was conducted at the Experimental Center of Shanghai University of Traditional Chinese Medicine. DSS (MP Biomedicals, USA) was dissolved in distilled water to a 5 mg/mL final concentration.

The UPLC system was performed on an Agilent 1290 LC system (Agilent Technologies Inc., Palo Alto, CA, USA) equipped with a binary pump. The mass spectrometer AB Sciex Triple TOF® 4600 (AB SCIEX, Foster City, CA, USA), equipped with electrospray ionization (ESI) source, was controlled by Analyst TF 1.7.1. software (AB SCIEX, Foster City, CA, USA). The spectrometer was operated in full-scan TOF-MS at *m*/*z* 50-1700 and information-dependent acquisition (IDA) MS/MS modes, with negative and positive ionization modes. Data analysis was performed using PeakView 1.2 software (AB SCIEX, Foster City, CA, USA).

**Figure 1:**
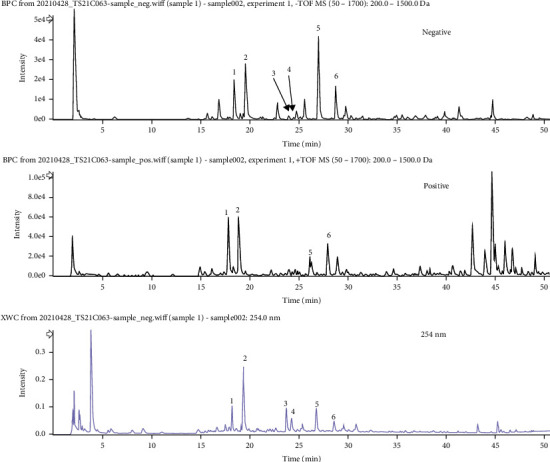
The base peak intensity chromatogram (negative/positive ion mode) and UV chromatogram (254 nm/280 nm/300 nm/330 nm) of KJT.

The major constituents of KJT were rapidly characterized using UPLC-Q/TOF-MS method in both positive and negative ion modes. Six compounds in KJT were unambiguously or tentatively characterized by comparing their retention times and MS/MS spectra with those in Natural Products HR-MS/MS Spectral Library database or published data [29–32]. The detailed compound information is summarized in Table 1, and the relevant chromatograms are displayed in Figure 1.

### 2.2. Animals and Ethics Statement

Forty males, six weeks of age, Sprague Dawley (SD) rats were purchased from Shanghai Xipur-Bikai Experimental Animal Co., Ltd. (Shanghai, China). All animals were housed in a controlled environment (temperature, 23°C ± 2°C; relative humidity, 40%–70%; lighting cycle, 12 h/d; 07:00–19:00 light), with free access to food and water. This study was approved by the Experimental Animal Welfare and Ethics Committee of Shanghai University of Traditional Chinese Medicine. The study protocols adhered to relevant laws and regulations of experimental animal welfare ethics. The ethics approval number is PZSHUTCM190322002.

### 2.3. Establishment of a UC Rat Model

A seven-day intragastric administration of 5% DSS in distilled water established a UC rat model. Daily observations were made of body weight, stool characteristics, mental state, hematochezia, and eating conditions. The study did not include any dead animals. After model completion, two rats were randomly selected from control and model groups and sacrificed separately. Colon tissue was removed, and DAI scores were compared to determine model success.

### 2.4. Animal Grouping and Intervention

After seven days of DSS treatment, rats were divided into four groups (*n* = 9 each group) and given intragastric administration twice a day of the following: control group (CG, 3 mL saline/day), UC model group (UG, 3 mL saline/day), KJT group (KG, 10 mL/kg/day), and sulfasalazine (SASP) group (SG, 0.5 g/kg/day). Body weight, stool characteristics, mental state, and hematochezia were observed daily. After seven days administration, rats were killed by intraperitoneal injection using 100-150 mg/kg of pentobarbital sodium.

### 2.5. Colon Tissue Histology

We separated colon tissue of four groups of rats (*n* = 9 each group) and dissected the colon along the longitudinal axis of intestine. The most damaged colon tissue (2 cm) was fixed in 4% paraformaldehyde for 24 h at -80°C and then embedded in paraffin. The sections were 5 *μ*m thick, stained with hematoxylin-eosin (H&E) and periodic acid-Schiff (PAS), and observed under a DP73 optical microscope (Olympus, Tokyo, Japan). Colon integrity, changes in colonic recesses, goblet cell distribution, and inflammatory cell infiltration were all observed and photographed.

### 2.6. Reverse Transcription-Quantitative PCR (RT-qPCR)

We used Trizol reagent (Invitrogen, USA) to extract mRNA from 100 mg colonic mucosa from each rat and reverse transcribed it into cDNA using a reverse transcription kit (Takara Bio, Japan). Primers were synthesized by Sangon Biotech Co. Ltd. (Shanghai, China) (Table 2). We used a One Step Plus Real-Time PCR instrument (Application Biosystems, USA) for RT-qPCR analysis of target genes. Amplification conditions were 95°C cycle, 10 min cycle, 95°C cycle, 15 s, 60°C cycle, 60 s, 30 rounds. We used the 2-^*ΔΔ*Ct^ method to calculate the relative expression of target mRNA and normalized it to GADPH.

### 2.7. Western Blot Analysis

Approximately 100 mg colonic tissue was ground down in liquid nitrogen. The total protein was then extracted using RIPA lysate buffer (Cell Signaling Technology, Inc., Danvers, MA, USA) supplemented with protease inhibitors (Bimake, Shanghai, China). The total protein was then quantified using a bicinchoninic acid kit (Pierce, USA) and a spectrophotometer (BioTek, Vermont, USA). Following that, proteins were loaded and electrophoresed on SDS-PAGE (4-20%, BBI Life Sciences, Shanghai, China) gels, transferred to polyvinylidene fluoride membranes, and blocked in Tris-buffered saline Tween (TBST, Beyotime Biotechnology, Shanghai, China) containing 5% skimmed milk at room temperature for 1 h. Primary antibodies were diluted, added to membranes, and incubated overnight at 4°C. The next day, membranes were washed four times in Phosphate Buffer Solution Tween-20 (PBST, Beyotime Biotechnology, Shanghai, China). After that, secondary antibodies were diluted, added to membranes, and incubated at room temperature for 1 h. An electrochemiluminescence (ECL) western blot system (Tanon 4200SF, Shanghai, China) highlighted protein bands. These were then analyzed using ImageJ software (Massachusetts, USA). Primary antibodies: anti-NEK7 (Abcam, ab133514, UK, 1 : 5000); anti-NLRP3 (Arigo, ARG40539, Taiwan, 1 : 500); anti-ASC (Abcam, ab151700, UK, 1 : 500); anti-caspase-1 (Arigo, ARG40539, Taiwan, 1 : 1000); anti-cleaved-caspase-1 (CST, 89332, USA, 1 : 1000); anti-GSDMD (CST, 93709, USA, 1 : 500); anti-GSDMD-N (Abcam, ab215203, UK, 1 : 500). Secondary antibodies: anti-mouse IgG (Arigo, ARG65350, Taiwan, 1 : 10000) and anti-rabbit IgG (Arigo, ARG65351, Taiwan, 1 : 10000).

### 2.8. Cytokine Analysis Using Enzyme-Linked Immunosorbent Assay (ELISA)

Whole blood (5–10 mL) from rat abdominal aorta was collected in dry test tubes. The serum was separated by centrifugation at 4000 rpm for 10 min and stored at -20°C. Colon tissue samples were rinsed with saline, homogenized with PBS, and centrifuged for 20 min at 3000 rpm/min. Following that, the supernatant was extracted for detection. IL-1*β*, IL-18, and IL-33 content of serum and colon tissue were analyzed using ELISA. All ELISA kits are purchased from mlbio Biotechnology Co., Ltd. (Shanghai, China).

### 2.9. Gut Microbiota Analysis

After the last administration, 3–5 grains of fresh feces were collected from each rat, placed into sterilized centrifuge tubes, and stored at -20°C for intestinal flora analysis. Using E.Z.N.A.® soil DNA kit (Omega BioTek, Norcross, GA, USA), fecal genomic DNA was extracted according to manufacturer's instructions. Using V3 region of 16s rRNA gene, the following universal primers were used for PCR amplification: 338F (5′-ACTCCTACGGGAGGCAGCAG-3′) and 806R (5′-GGACTACHVGGGTWTCTAAT-3′). DNA integrity was detected by 1% agarose gel electrophoresis (voltage 5 V/cm, 20 min). The concentration of DNA was measured by NanoDrop 2000 and the loading concentration of DNA was greater than 5 ng/*μ*L. Sequencing was performed using Illumina's MiSeq PE300 platform and nucleotide length was 468 bp.

Quality control of the raw sequenced sequences was performed using fastp (https://github.com/OpenGene/fastp, version 0.20.0) software to enable FLASH (http://www.cbcb.umd.edu/software/flash, version 1.2.7) software to perform splicing. (1) Filter the bases below 20 mass value at the tail of reads, set a window of 50 bp, truncate the back-end bases from the window if the average mass value within the window is below 20, filter the reads below 50 bp after quality control, and remove the reads containing N bases. (2) Splice (merge) pairs of reads into one sequence based on the overlap relationship between PE reads, with a minimum overlap length of 10 bp. (3) The maximum mismatch ratio allowed in the overlap region of the spliced sequence is 0.2, screening out nonconforming sequences. (4) Distinguish samples according to the barcode and primers at the beginning and end of the sequence, and adjust the sequence orientation, the number of mismatches allowed for barcode is 0, and the maximum number of primer mismatches is 2.

### 2.10. Bioinformatics Analysis

Using the UPARSE software (http://drive5.com/uparse/, version 7.1), the sequences were OTU clustered based on a 97% similarity, as follows. (1) Extract nonrepetitive sequences from the optimized sequences and remove single sequences without duplicates. (2) OTU clustering of nonrepetitive sequences (without single sequences) according to 97% similarity, removing chimeras in the clustering process to obtain OTU representative sequences. (3) Map all optimized sequences to OTU representative sequences, select sequences with 97% or more similarity to OTU representative sequences, and generate OTU tables.

Each sequence was annotated for species classification using RDP classifier (http://rdp.cme.msu.edu/, version 2.2), compared to the Silva 16S rRNA database (v138), and a comparison threshold of 70% was set.

Statistical analysis was performed using R (version 3.3.1). The gut microbial *β*-diversity analysis (PCoA analysis) and PLS-DA analysis were used to compare the fecal microbiota of each group. The bar map of intestinal flora at each classification level (phylum, class, order, family, genus, and species) and the heatmaps of intestinal flora at family, genus, and species level were drawn to display the composition and species abundance information of intestinal flora in each group. Linear discriminant analysis effect size (LEfSE) was used to analyze the species characteristics and differences of intestinal flora in each group.

### 2.11. Statistical Analyses

All data were expressed as mean ± standard deviation (SD). SPSS 21.0 software (IBM, New York, USA) was used for statistical analysis, and variables between groups were analyzed using one-way single-factor analysis of variance (ANOVA) in conjunction with Tukey HSD post hoc test. Differences were statistically significant at *P* < 0.05.

## 3. Results

### 3.1. The Effects of KJT on Body Weight, Colon Length, and DAI Scores in UC Rats

**Figure 2:**
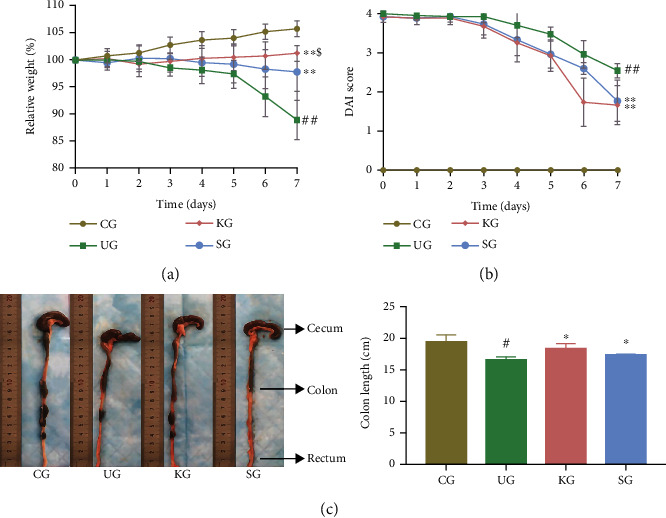
The effects of KJT in UC rats. (a) Changes in body weight. (b) Changes in DAI scores. (c) Rat colon length. *N* = 9 animals/group. Values are expressed as the mean ± standard deviation (SD); ^#^*P* < 0.05 and ^##^*P* < 0.001 vs. CG; ^∗^*P* < 0.05 and ^∗∗^*P* < 0.001 vs. UG; ^$^*P* < 0.05 vs. UG.

After successfully establishing the UC rat model, body weight, colon length, and DAI scores were used to evaluate KJT efficacy for UC. During intervention, rat body weight in each group was measured daily. Rat body weight increased daily in CG. In comparison, body weight in UG animals was significantly decreased on day six (Figure 2(a), *P* < 0.001). Following KJT and SASP intervention, KG and SG had significantly higher body weights than UG (Figure 2(a), *P* < 0.001). In addition, the rats in the KG group were heavier than those in the SG group (Figure 2(a), *P* < 0.05).

On the 7^th^ day of intervention, UG, KG, and SG rats had significantly higher DAI scores than CG rats (Figure 2(b), *P* < 0.001), and these scores decreased gradually over time during the intervention period. When KG and SG rats were compared to UG rats, their DAI scores were significantly lower (Figure 2(b), *P* < 0.001), without observing a significant difference between KG and SG (Figure 2(b), *P* > 0.05). Colon length in each group was measured on the 7^th^ day of intervention. Colon length was significantly shorter in UC rats than in CG rats (Figure 2(c), *P* < 0.05). However, this length increased after intervention with SASP and KJT (Figure 2(c), *P* < 0.05), without observing a significant difference between the two groups (Figure 2(c), *P* > 0.05).

### 3.2. The Effects of KJT on Colonic Mucosa in UC Rats

**Figure 3:**
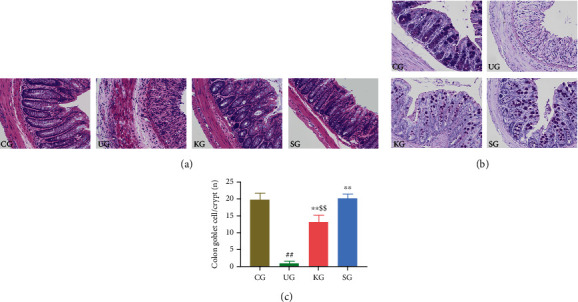
Pathological manifestations of colon tissue in rats. (a) H&E staining of colon tissues of rats in each group (×200 magnification). (b) PAS staining of colon tissues of rats in each group (×200 magnification). (c) The number of goblet cells in colon tissue of rats in each group. *N* = 9 animals/group. ^##^*P* < 0.001 vs. CG; ^∗∗^*P* < 0.001 vs. UG; ^$$^*P* < 0.05 vs. SG.

The histopathological data revealed that colonic villi of CG rats were morphologically intact, the epithelial structure of colonic mucosa was clearly visible, and multiple cells were neatly arranged in intestinal glands without any lesions. The epithelial structure in colon tissue of UG rats was completely lost, crypts and goblet cells disappeared, and inflammatory cell infiltration was increased. In contrast, after KJT intervention, these pathological manifestations were improved, with essentially intact mucosal structures and a significantly increased number of goblet cells. SG rats had a similar colonic pathology to CG rats (Figures 3(a)–3(c)). These findings suggested that KJT protected the colonic mucosa of UC rats.

### 3.3. The Effects of KJT on the NEK7-NLRP3/Caspase-1/GSDMD Signaling Pathway in UC Rats

**Figure 4:**
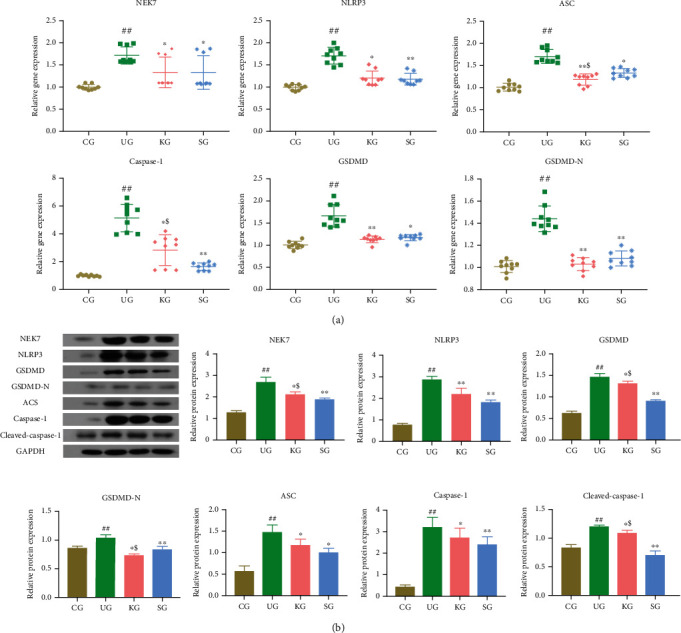
NEK7, NLRP3, ASC, caspase-1, cleaved-caspase-1, GSDMD, and GSDMD-N relative mRNA and protein levels in the colonic tissue of rats. (a) Relative mRNA expression in rat groups. (b) Relative protein expression in rat groups. *N* = 9 animals/group. Values are expressed as the mean ± standard deviation (SD). ^##^*P* < 0.001 vs. CG; ^∗^*P* < 0.05 and ^∗∗^*P* < 0.001 vs. UG; ^$^*P* < 0.05 vs. UG.

To further elucidate KJT anti-inflammatory mechanisms, we used RT-qPCR and western blotting to assess relative mRNA and protein expression of NEK7, NLRP3, ASC, caspase-1, and GSDMD in colon tissues from study rats. When compared with CG, the relative mRNA and protein expression of NEK7, NLRP3, ASC, caspase-1, and GSDMD in colonic tissue from UG rats was significantly increased (Figures 4(a) and 4(b), *P* < 0.001), implying that DSS successfully promoted pyroptosis, leading to UC, by activating NEK7 and NLRP3 inflammasome. After SASP and KJT treatment, the relative mRNA and protein expression of NEK7, NLRP3, ASC, caspase-1, and GSDMD decreased significantly compared with UG (Figures 4(a) and 4(b), *P* < 0.05). Among them, compared with the SG group, the KG group had lower relative mRNA expression of ASC (Figure 4(a), *P* < 0.05), while the relative mRNA expression of caspase-1 was higher (Figure 4(a), *P* < 0.05). In addition, the KG group had higher relative protein expression of NEK7, GSDMD, and cleaved-caspase-1 (Figure 4(b), *P* < 0.05), whereas the relative protein expression of GSDMD-N was lower (Figure 4(b), *P* < 0.05). These data indicated that KJT successfully ameliorated UC by inhibiting NEK7 activation, which is an activator protein of NLRP3 inflammasome.

### 3.4. The Effects of KJT on Proinflammatory Factors in UC Rats

**Figure 5:**
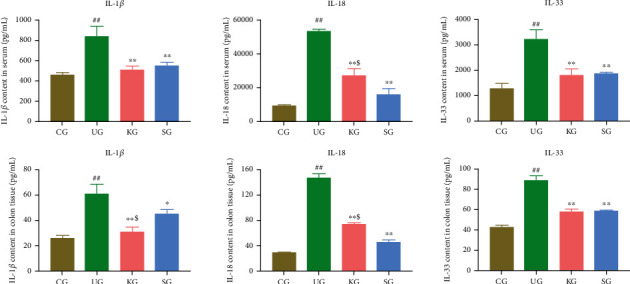
The contents of proinflammatory factors in serum and colon tissue of rats. *N* = 9 animals/group. Values are expressed as the mean ± standard deviation (SD). ^##^*P* < 0.001 vs. CG; ^∗^*P* < 0.05 and ^∗∗^*P* < 0.001 vs. UG; ^$^*P* < 0.05 vs. UG.

Because IL-1*β*, IL-18, and IL-33 are proinflammatory factors that contribute to pyroptosis, their serum and colon tissue levels in rats in each group were measured. Compared with CG, rats of UG had significantly higher levels of IL-1*β*, IL-18, and IL-33 in their serum and colon tissues (Figure 5, *P* < 0.001). After treatment with KJT and SASP, the levels of IL-1*β*, IL-18, and IL-33 in serum and colon tissues were significantly decreased (Figure 5, *P* < 0.001). The contents of IL-18 in colon tissues and serum of rats in the KG group were significantly higher than those in the SG group (Figure 5, *P* < 0.05), whereas the contents of IL-1*β* in colon tissues of rats in the KG group were significantly lower than those in the SG group (Figure 5, *P* < 0.05). These data indicated that KJT exerted adequate anti-inflammatory effects.

### 3.5. KJT Promotes Intestinal Homeostasis in UC Rats

**Figure 6:**
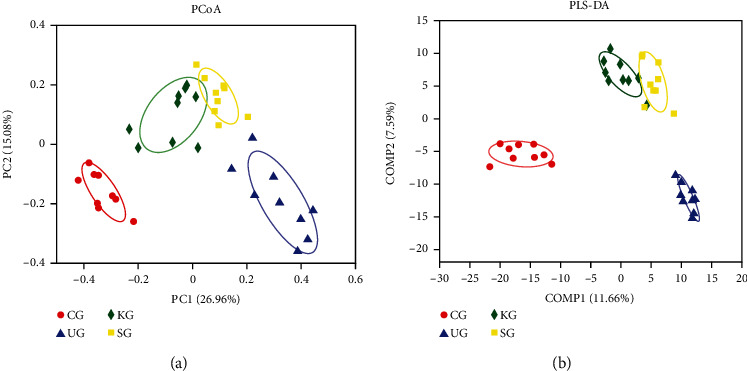
The gut microbial *β*-diversity analysis and PLS-DA of fecal microbiota of each group. (a) PCoA plot. (b) PLS-DA plot. *N* = 9 animals/group.

To assess gut microbial community composition structure in each group of rats, we used *β*-diversity analysis and Partial Least Squares Discriminant Analysis (PLS-DA), respectively. The microbial community structure of all groups had obvious clustering characteristics, in which the microbial community structure of rats in CG and UG was significantly different. In contrast, the microbial community structure of rats in KG and SG was more similar to that of CG (Figures 6(a) and 6(b)).

**Figure 7:**
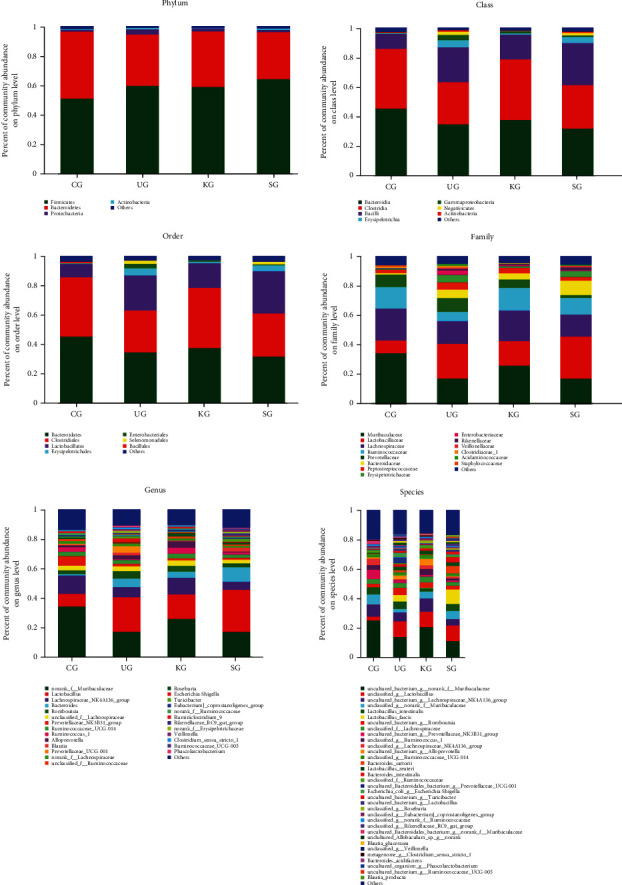
Histograms showing relative bacterial abundance. *N* = 9 animals/group.

**Figure 8:**
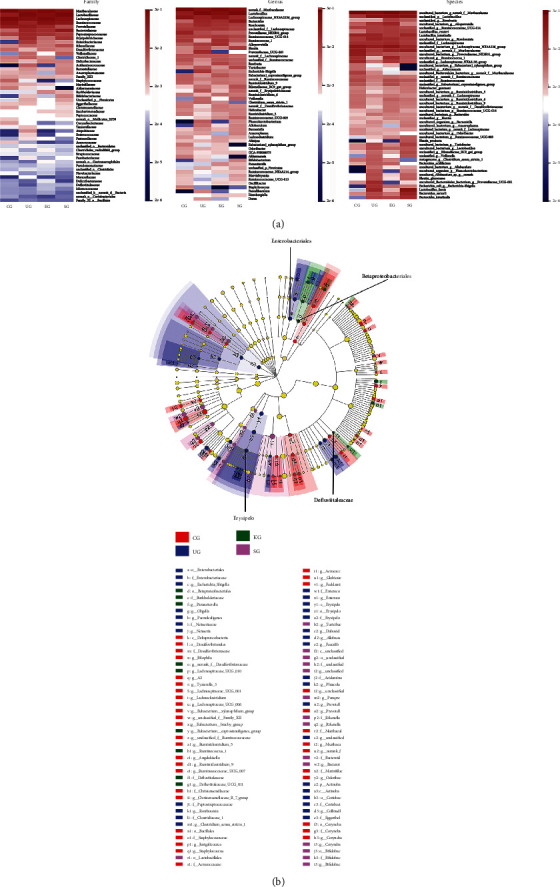
The relative abundance of microbiota in rat feces. (a) Heatmaps showing the relative abundance of microorganisms. (b) Linear discriminant analysis effect size (LEfSE) of the relative abundance of microorganisms. *N* = 9 animals/group.

Additionally, our data revealed the community structure of intestinal microflora and the relative abundance of major intestinal microorganisms at phylum, class, order, family, genus, and species levels (Figure 7, Table S1). We also used our data to generate heatmaps illustrating the relative abundance of microorganisms in each group at the most representative classification level (family, genus, and species) (Figure 8(a)).

Compared with CG feces, the relative abundance of Erysipelotrichia, Erysipelotrichales, Erysipelotrichaceae, and Turicibacter in UG feces increased significantly (Table S1, *P* < 0.05). After KJT treatment, these bacteria were significantly reduced (Table S1, *P* < 0.05). In addition, the relative abundance of Ruminococcaceae was significantly lower in UG rats than in CG rats (Table S1, *P* < 0.05) but significantly increased in KG rats (Table S1, *P* < 0.05).

Additionally, we used linear discriminant analysis (LDA) to identify distinct species between groups. Comparing UG and KG relative abundances revealed differences in consistency at the order (Enterobacteriales and Betaproteobacteriales) and family (Erysipelo and Defluviitaleaceae) levels (Figure 8(b)). These findings indicated that KJT effectively corrected intestinal flora disorders in UC rats.

## 4. Discussion

UC is an inflammatory bowel disease characterized by flora imbalance, abnormal activation of innate immunity, and interactions between these factors [33, 34]. It is accepted that NLRP3-like receptors, as an innate immune receptor [35, 36], trigger immune and inflammatory responses after infection. NLRP3 inflammatory bodies are representative molecules of this family. In UC animal models (induced by DSS), NLRP3 expression is increased [37], but the lack of NLRP3 is not sensitive to DSS modeling [38]. Initiating NLRP3 depends on transcriptional activation of NF-*κ*B. NEK7 is an NLRP3 activating protein, a serine or threonine kinase, expressed in heart, brain, liver, and other tissues and is a key enzyme involved in pyroptosis. Regarding NLRP3 regulation by NEK7, studies have confirmed interactions between NEK7 and NLRP3 in mice and cell models using coimmunoprecipitation (Co-IP) and glutathione-S-transferase (GST) pull-down approaches [39]. Additionally, transcription factor p65 has been suggested to activate NEK7 transcription by targeting NEK7 promoter region and participating in NLPR3 activation. NLRP3 is then activated by stimuli such as potassium ion outflow, reactive oxygen species, crystals, or particles entering the cell. NEK7 forms the NEK7-NLRP3 complex by specifically binding to NLRP3 leucine repeat (LRR) and NLR characteristic domain NACHT [15]. NEK7 deletion specifically inhibits NLRP3 activation and reduces the expression of downstream caspase-1 and cleaved-caspase-1, thereby reducing downstream inflammatory factor secretion. Additionally, when the NEK7-NLRP3 inflammatory complex is assembled and activated, the effector protein caspase-1, in addition to conventional splicing inflammatory factor precursors IL-1*β*, IL-18, and IL-33, specifically cuts a key protein called Gasdermin-D (GSDMD) [40], exposing its N-terminal domain (GSDMD-N), which then destroys the cell membrane composed of specific phospholipids and polymerizes to form regular pores on the lipid membrane. In turn, this process mediates the secretion and release of numerous mature inflammatory factors, followed by osmotic swelling and pyroptosis.

In this study, seven days after UC model establishment, the body weight of UG animals was decreased, whereas DAI scores decreased slowly. These data were significantly different from those obtained with CG animals. The KJT (KG) and SASP groups (SG) revealed increased body weight to varying degrees, and these weights were significantly higher than those of UG animals. UG animals had significantly shorter colon lengths than CG animals, without differences between the KG, SG, and CG groups. Regarding colonic pathology, UG rats had disordered epithelium structures, intestinal recess and goblet cells had disappeared, the submucosa was thickened, and the epithelium was accompanied by inflammatory cell infiltration. However, following KJT administration, this pathology improved significantly; colonic mucosa structure remained intact, goblet cells increased, and inflammatory cell infiltration decreased, similar to SG and CG animals. These findings indicated that KJT alleviated inflammatory injuries mediated by UC.

We then used RT-qPCR and western blotting to determine the expression of NEK7, NLRP3, ASC, caspase-1, cleaved-caspase-1, GSDMD, and GSDMD-N mRNA or protein. We observed that UG animals had significantly higher mRNA and protein levels of key pyroptosis molecules than CG animals. After KJT and SASP administration, NEK7, NLRP3, ASC, caspase-1, cleaved-caspase-1, GSDMD, and GSDMD-N mRNA and protein levels were significantly lower than those in UG. In addition, the contents of IL-1*β*, IL-18, and IL-33 in intestinal mucosa and serum were significantly lower than those in UG. The above results suggest that KJT can further block UC occurrence and protect UC rats by inhibiting the transcription and protein expression of NEK7-NLRP3 inflammatory body complex.

It is known that pyroptosis is closely related to the development of UC diseases, in which overactivation of the NLRP3/caspase-1 classical pyroptosis pathway may play a key role [41]. Firstly, when stimulated by oxidative stress-induced damage signals such as ATP and mitochondrial DNA or by viral or bacterial infections [42], NLRP3 has the ability to promote the formation of inflammatory vesicles and the activation of MAPK and NF-*κ*B signaling cascade response activation, initiating and supporting the immune response [43]. In contrast, macrophages lacking NLRP3 do not have IL-1*β* secretion [44], and inhibition of Caspase-1 with prasugan can achieve a level of mucosal protection comparable to that of NLRP3 deficiency. It was demonstrated that expression of caspase-1, NLRP3, and GSDMD was significantly higher in human inflammatory bowel tissue compared to normal bowel tissue. Increased production of IL-1*β* and IL-18 in the intestinal mucosa of patients with active UC and increased caspase-1 activity in intestinal tissues and macrophages were found [45]. Caspase-1 activates IL-1*β* and IL-18 while accelerating the release of inflammatory factors, which are not activated after knocking out the caspase-1 gene in mice.

In the present study, the expression of NEK7, NLRP3, ASC, caspase-1, cleaved-caspase-1, GSDMD, and GSDMD-N mRNA and protein in colonic tissues was significantly inhibited by KJT intervention in post-DSS mice, while at the same time it could differentially reduce the expression of IL-1*β*, IL-18, and IL-33 levels in colon tissue and serum; i.e., the KJT side could negatively regulate NLRP3 activation, assembly, cell swelling necrosis, and the secretion and release of inflammatory factors at all levels, thereby reducing the inflammatory damage of UC.

While UC etiology and pathogenesis are currently unclear, UC pathogenesis may also be closely associated with host intestinal flora disorder; thus, an imbalanced intestinal flora may initiate and promote factors underpinning this particular UC condition [35, 46]. NLRP3 inflammasome can interact with many downstream signaling pathways and is critical for intestinal microecology regulation. Intestinal flora imbalances can also activate NLRP3 inflammasome, exacerbating intestinal inflammatory damage [47, 48]. Our study found that UG rats increased Lactobacillales, Lactobacillaceae, Lactobacillus, unclassified_g__Lactobacillus, Erysipelotrichia, Erysipelotrichales, Erysipelotrichaceae, Turicibacter, and uncultured_bacterium_g__Turicibacter and decreased Bacteroidetes, Bacteroidia, Bacteroidales, Ruminococcaceae, unclassified_f__Ruminococcaceae, Ruminiclostridium_9, unclassified_g__Ruminococcus_1, unclassified_f__Ruminococcaceae, and unclassified_g__Lachnospiraceae_NK4A136_group, compared with CG. These results were similar to another study comparing fecal intestinal flora in UC patients and healthy individuals, which found that Bacteroidaceae, Bacteroides, Firmicutes, and Clostridia were dominant in healthy individuals. In contrast, Lactobacillus, Lactobacillaceae, Erysipelotrichaceae, and Erysipelotrichales were dominant in UC patients [49]. Additionally, a recent study concluded that Ruminococcus and Lachnospiraceae were key flora in Crohn's disease (CD) and UC and that Bacteroidaceae decreased primarily in UC patients [50]. Although the link between Turicibacter and inflammatory bowel disease (IBD) has not been established conclusively, some researchers believe it is directly related to DSS modeling [51]. Turicibacter is a proinflammatory bacterium closely related to Alzheimer's disease, and its presence indicates brain inflammation and is associated with increased levels of inflammatory mediators in patients with cognitive impairment and cerebral amyloidosis [52]. In UC rats intervened by KJT, we observed that KJT increased Ruminococcaceae, unclassified_f__Ruminococcaceae, and unclassified_g__Ruminococcus_1 levels but decreased Erysipelotrichia, Erysipelotrichales, Erysipelotrichaceae, Turicibacter, and uncultured_bacterium_g__Turicibacter levels. Ruminococcus is an important bacterial group in UC. In a comparison study of microbial compositions of patients with collagenous enteritis (GC), CD, and UC, it was found that of ten operational taxonomic units (OTU) observed in CG patients with a decrease in Ruminococcus family, nine OTU were reduced in CD patients, while four out were reduced in UC patients [53]. Another study indicated that Ruminococcus was a mainstream flora that converted primary bile acids in the intestine into secondary bile acids [54]; however, Ruminococcus abundance in UC patients is insufficient, resulting in secondary bile acid deficiency, thus promoting the proinflammatory state in intestinal tract. As a result, KJT appears to regulate part of intestinal flora to facilitate UC improvements.

Thanks to intestinal flora differences between IBD patients and healthy individuals, bacterial transplantation may have treatment implications for certain patient groups [55]. Numerous clinical trials investigating fecal bacteria transplantation are currently underway [56–58]. Regarding UC, a small series of Re-FMT cases for moderate and severe UC reported that FMT could induce remission in patients with moderate to severe UC, but half of patients recurred at 54 weeks, and the curative effect of Re-FMT was worse than that of the first treatment, but the disease was milder than before at the time of recurrence [59]. Consistent with another study's findings, it was reported that the response of UC patients to FMT was transient [60]. Other studies also used FMT to treat E2-active UC patients and compared microflora changes between 5-aminosalicylic acid (5-ASA) and FMT. It was observed that 5-ASA did not affect changes in intestinal flora. Although the relative abundance of fecal Bifidobacterium, Bacillus, Lactobacillaceae, Rumen coccidiaceae, and Clostridium increased significantly in clinical remission patients following FMT, researchers believe that increased intestinal beneficial groups are insufficient for UC remission. The effectiveness of FMT has indicated that intestinal flora is an important regulatory factor for IBD. Future research on intestinal flora and fecal bacteria transplantation should be more comprehensive.

## 5. Conclusions

We observed that KJT restored body weight and colon length in a DSS-induced UC rat model. KJT decreased DAI scores, ameliorated pathological injury to the colon, and inhibited signal molecules involved in the inflammatory NEK7-NLRP3/caspase-1 pathway, as well as other downstream inflammatory factors. Similarly, KJT appeared to regulate intestinal flora. These processes underpin UC remission mechanisms induced by KJT.

## Figures and Tables

**Table 1:** Identification of the major chemical compounds in KJT.

No.	RT (min)	Measured *m*/*z*	Respected *m*/*z*	ppm	Formula	M.W.	Identification	MS/MS data
1	18.47	449.1314	449.1301	3	C_17_H_24_O_11_	404.13	Hastatoside	449.1277; 241.0710; 223.0605; 209.0467; 195.0657
2	19.62	433.1353	433.1352	0.3	C_17_H_24_O_10_	388.14	Verbenalin	433.2065; 387.2025; 293.1297; 225.0776; 193.0518
3	24.01	300.9988	300.999	-0.6	C_14_H_6_O_8_	302.01	Ellagic acid	300.9957; 284.0051; 201.0197; 145.0296; 117.0350
4	24.51	193.051	193.0506	1.9	C_10_H_10_O_4_	194.06	Ferulic acid	193.0503; 178.0278; 134.0371
5	27.04	623.1993	623.1981	1.9	C_29_H_36_O_15_	624.21	Isoacteoside	623.1624; 315.0500; 161.0248
6	28.85	609.1839	609.1825	2.3	C_28_H_34_O_15_	610.19	Hesperidin/neohesperidin	609.1838; 301.0713; 286.0477; 242.0562

**Table 2:** Primers for qRT-PCR.

Name	Primer sequence
NEK7	Sense: 5′-AGGCCTTACGACCGGATATG-3′
NEK7	Antisense: 5′-TCCATCCAAGAGACAGGCTG-3′
NLRP3	Sense: 5′-CCCAGGGATGAGAGTGTTGT-3′
NLRP3	Antisense: 5′-CAAGGAGATGTCGAAGCAGC-3′
ASC	Sense: 5′-TCTACCTGGAGACCTACGGC-3′
ASC	Antisense: 5′-TCCAGAGCCCTGGTGC-3′
Caspase-1	Sense: 5′-GAAAAGCCATGGCCGACAAG-3′
Caspase-1	Antisense: 5′-GCCCCTTTCGGAATAACGGA-3′
GSDMD	Sense: 5′-CAGTTTCACTTTTAGCTCTGGGC-3′
GSDMD	Antisense: 5′-CCCATGCTCCGTGACCG-3′
GSDMD-N	Sense: 5′-TGAATGTGTACTCGCTGAGTGTGG-3′
GSDMD-N	Antisense: 5′-CAGCTGCTGCAGGACTTTGTC-3′
GAPDH	Sense: 5′-CCATCACCATCTTCCAGG-3′
GAPDH	Antisense: 5′-ATGAGTCCTTCCACGATAC-3′

## Data Availability

The datasets generated and/or analyzed during the current study are not publicly available due to patent applications but are available from the corresponding author on reasonable request.

## Supplementary Materials

Supplementary Materials Table S1: the relative abundance of intestinal microflora in the six classification levels of feces of rats in each group.
