# Supplementary material for: Kui Jie Tong Ameliorates Ulcerative Colitis by Regulating Gut Microbiota and NLRP3/Caspase-1 Classical Pyroptosis Signaling Pathway
Source: Dis Markers. 2022 Jul 4;2022:2782112. doi: 10.1155/2022/2782112 (PMC9273439; doi:10.1155/2022/2782112)
Supplement: Supplementary Materials — Table S1: the relative abundance of intestinal microflora in the six classification levels of feces of rats in each group. [file 2782112.f1.doc]

| Table S1. The relative abundance of intestinal microflora in the six classification levels of faeces of rats in each group. | | | | | |
| --- | --- | --- | --- | --- | --- |
| Categoy | Taxonomic group | CG | UG | KG | SG |
| Phylum |  |  |  |  |  |
|  | Firmicutes | 51.03±7.87 | 59.74±10.02 | 59.10±14.01 | 64.22±7.71 |
|  | Bacteroidetes | 45.38±10.06 | 34.15±6.74^#^ | 37.21±13.59 | 31.71±8.10 |
|  | Proteobacteria | 1.09±0.28 | 3.88±7.34 | 2.28±1.80 | 1.51±1.35 |
|  | Actinobacteria | 0.62±1.25 | 1.15±1.70 | 0.21±0.11 | 0.93±1.14 |
| Class |  |  |  |  |  |
|  | Bacteroidia | 45.38±10.06 | 34.15±6.74^#^ | 37.21±13.59 | 31.71±8.10 |
|  | Clostridia | 40.72±7.97 | 28.85±11.84 | 41.43±10.54 | 28.63±17.85 |
|  | Bacilli | 9.70±7.17 | 23.29±14.55 | 16.51±7.18 | 29.53±20.30 |
|  | Erysipelotrichia | 0.55±0.52 | 4.86±4.32^#^ | 0.92±0.48^*^ | 3.95±2.69 |
|  | Gammaproteobacteria | 0.12±0.05 | 3.61±7.43 | 1.44±1.49 | 0.92±1.36 |
|  | Negativicutes | 0.02±0.01 | 2.44±4.03 | 0.09±0.12 | 1.74±3.44 |
|  | Actinobacteria | 0.62±1.25 | 1.15±1.70 | 0.21±0.11 | 0.93±1.14 |
| Order |  |  |  |  |  |
|  | Bacteroidales | 45.30±10.08 | 34.13±6.74^#^ | 37.18±13.60 | 31.70±8.09 |
|  | Clostridiales | 40.72±7.97 | 28.85±11.84 | 41.43±10.54 | 28.63±17.85 |
|  | Lactobacillales | 8.64±6.45 | 23.20±14.56^#^ | 16.50±7.18 | 29.50±20.30 |
|  | Erysipelotrichales | 0.55±0.52 | 4.86±4.32^#^ | 0.92±0.48^*^ | 3.95±2.69 |
|  | Enterobacteriales | 0.00±0.00 | 3.30±7.37 | 0.75±1.28 | 0.59±1.30 |
|  | Selenomonadales | 0.02±0.01 | 2.44±4.03 | 0.10±0.12 | 1.74±3.44 |
|  | Bacillales | 1.06±2.37 | 0.09±0.06 | 0.01±0.01^*^ | 0.03±0.02^*^ |
| Family |  |  |  |  |  |
|  | Muribaculaceae | 34.46±11.75 | 17.03±5.86^#^ | 25.88±15.87 | 17.26±9.28 |
|  | Lactobacillaceae | 8.37±6.40 | 22.93±14.83^#^ | 16.43±7.17 | 29.310±20.230 |
|  | Lachnospiraceae | 22.02±7.95 | 16.24±11.50 | 21.04±7.49 | 14.470±11.280 |
|  | Ruminococcaceae | 14.64±4.20 | 6.07±4.20^#^ | 15.25±5.02^*^ | 10.910±7.389 |
|  | Prevotellaceae | 8.60±8.50 | 8.62±7.76 | 5.65±9.89 | 1.901±2.314 |
|  | Bacteroidaceae | 1.05±0.70 | 6.23±8.64 | 4.23±1.98 | 9.8374.543 |
|  | Peptostreptococcaceae | 2.36±3.22 | 4.94±2.14 | 3.86±1.76 | 2.602±1.623^*^ |
|  | Erysipelotrichaceae | 0.55±0.52 | 4.86±4.32^#^ | 0.92±0.48^*^ | 3.945±2.694 |
|  | Enterobacteriaceae | 0.00±0.00 | 3.30±7.37 | 0.75±1.28 | 0.586±1.300 |
|  | Rikenellaceae | 0.50±0.28 | 1.15±1.38 | 0.81±0.61 | 1.868±1.087 |
|  | Veillonellaceae | 0.02±0.01 | 1.12±2.46 | 0.09±0.12 | 1.288±3.264 |
|  | Clostridiaceae_1 | 0.66±0.86 | 1.09±0.76 | 0.52±0.42 | 0.010±0.008^*^ |
|  | Acidaminococcaceae | 0.00±0.00 | 1.32±2.06 | 0.00±0.00 | 0.448±0.952 |
|  | Staphylococcaceae | 1.05±2.36 | 0.06±0.05 | 0.00±0.00^*^ | 0.023±0.014 |
| Genus |  |  |  |  |  |
|  | norank_f__Muribaculaceae | 34.42±11.75 | 17±5.86^#^ | 25.88±15.88 | 17.26±9.28 |
|  | Lactobacillus | 8.37±6.40 | 22.93±14.83^#^ | 16.43±7.17 | 29.31±20.23 |
|  | Lachnospiraceae_NK4A136_group | 12.77±7.02 | 7.11±9.61 | 11.46±4.11 | 5.42±7.34 |
|  | Bacteroides | 1.05±0.70 | 6.23±8.64 | 4.23±1.98 | 9.84±4.54 |
|  | Romboutsia | 2.35±3.21 | 4.90±2.12 | 3.84±1.75 | 2.59±1.61^*^ |
|  | unclassified_f__Lachnospiraceae | 3.17±0.88 | 3.48±2.81 | 3.82±2.09 | 2.29±2.22 |
|  | Prevotellaceae_NK3B31_group | 6.46±8.36 | 1.79±2.31 | 1.23±2.52 | 1.16±1.98 |
|  | Ruminococcaceae_UCG-014 | 2.65±1.63 | 2.32±3.12 | 3.08±1.63 | 2.12±1.69 |
|  | Ruminococcus_1 | 3.38±3.46 | 0.34±0.33 | 4.03±2.92^*^ | 1.57±2.70 |
|  | Alloprevotella | 1.35±0.92 | 2.53±2.97 | 4.38±10.2 | 0.49±1.18 |
|  | Blautia | 0.61±0.60 | 1.94±2.48 | 0.61±0.41 | 2.67±2.98 |
|  | Prevotellaceae_UCG-001 | 0.65±1.01 | 4.19±7.26 | 0.00±0.01 | 0.15±0.38 |
|  | norank_f__Lachnospiraceae | 1.49±0.69 | 1.35±0.94 | 1.34±0.74 | 0.84±1.07 |
|  | unclassified_f__Ruminococcaceae | 1.63±0.79 | 0.52±0.41^#^ | 1.47±0.21^*^ | 1.11±1.17 |
|  | Roseburia | 1.03±0.63 | 0.39±0.47 | 2.00±3.33 | 1.16±1.69 |
|  | Escherichia-Shigella | 0.00±0.00 | 3.30±7.37 | 0.75±1.27 | 0.58±1.30 |
|  | Turicibacter | 0.44±0.47 | 1.41±0.92^#^ | 0.42±0.23^*^ | 1.99±1.11 |
|  | Eubacterium_coprostanoligenes_group | 1.27±0.94 | 0.44±0.43 | 1.49±0.88^*^ | 0.82±0.41 |
|  | norank_f__Ruminococcaceae | 1.29±0.65 | 0.64±0.51 | 0.81±0.57 | 0.65±0.98 |
|  | Ruminiclostridium_9 | 1.13±0.37 | 0.39±0.41^#^ | 0.89±0.43 | 0.71±0.99 |
|  | Rikenellaceae_RC9_gut_group | 0.21±0.15 | 0.82±1.17 | 0.31±0.21 | 1.61±1.09 |
|  | norank_f__Erysipelotrichaceae | 0.10±0.11 | 1.48±1.71 | 0.21±0.18 | 0.99±1.46 |
|  | Veillonella | 0.02±0.01 | 1.12±2.46 | 0.09±0.12 | 1.29±3.26 |
|  | Clostridium_sensu_stricto_1 | 0.66±0.86 | 1.09±0.76 | 0.52±0.42 | 0.01±0.01^*^ |
|  | Ruminococcaceae_UCG-005 | 0.10±0.05 | 0.21±0.28 | 0.48±0.72 | 0.95±2.36 |
|  | Phascolarctobacterium | 0.00±0.00 | 1.32±2.06 | 0.00±0.01 | 0.45±0.95 |
| Species |  |  |  |  |  |
|  | uncultured_bacterium_g__norank_f__Muribaculaceae | 25.36±8.98 | 13.91±6.87^#^ | 20.74±14.76 | 11.53±6.19 |
|  | unclassified_g__Lactobacillus | 2.592±2.66 | 10.55±6.79^#^ | 10.57±5.99 | 11.2±10.86 |
|  | uncultured_bacterium_g__Lachnospiraceae_NK4A136_group | 8.20±5.23 | 6.44±8.67 | 8.90±2.92 | 4.19±6.33 |
|  | unclassified_g__norank_f__Muribaculaceae | 6.89±3.17 | 2.38±1.52^#^ | 4.54±2.46 | 5.51±5.90 |
|  | Lactobacillus_intestinalis | 4.72±3.78 | 4.69±5.98 | 2.37±3.13 | 4.30±5.13 |
|  | Lactobacillus_faecis | 0.03±0.02 | 4.24±5.98 | 0.21±0.37 | 10.37±15.43 |
|  | uncultured_bacterium_g__Romboutsia | 2.33±3.21 | 4.84±2.11 | 3.74±1.72 | 2.55±1.60^*^ |
|  | unclassified_f__Lachnospiraceae | 3.17±0.88 | 3.48±2.81 | 3.82±2.09 | 2.29±2.22 |
|  | uncultured_bacterium_g__Prevotellaceae_NK3B31_group | 6.42±8.36 | 1.73±2.24 | 1.23±2.52 | 1.16±1.98 |
|  | unclassified_g__Ruminococcus_1 | 3.38±3.46 | 0.23±0.27^#^ | 4.01±2.92^*^ | 1.30±2.04 |
|  | unclassified_g__Lachnospiraceae_NK4A136_group | 4.35±3.35 | 0.67±1.00^#^ | 2.56±2.27 | 1.22±1.70 |
|  | uncultured_bacterium_g__Alloprevotella | 1.06±0.95 | 2.52±2.97 | 4.37±10.17 | 0.49±1.18 |
|  | unclassified_g__Ruminococcaceae_UCG-014 | 1.97±1.43 | 1.88±2.66 | 2.26±1.06 | 1.57±1.26 |
|  | Bacteroides_sartorii | 0.44±0.27 | 1.69±3.08 | 0.63±0.48 | 5.41±4.43 |
|  | Lactobacillus_reuteri | 0.59±0.44 | 2.05±2.51 | 2.41±2.01 | 2.19±2.13 |
|  | Bacteroides_intestinalis | 0.01±0.01 | 1.98±4.22 | 1.66±0.97 | 3.34±2.96 |
|  | unclassified_f__Ruminococcaceae | 1.63±0.79 | 0.52±0.41^#^ | 1.47±0.21 | 1.11±1.17 |
|  | uncultured_Bacteroidales_bacterium_g__Prevotellaceae_UCG-001 | 0.42±0.71 | 3.52±7.34 | 0.00±0.01 | 0.13±0.35 |
|  | Escherichia_coli_g__Escherichia-ShigDorea_sp._5-2ella | 0.00±0.00 | 3.30±7.37 | 0.75±1.27 | 0.58±1.3 |
|  | uncultured_bacterium_g__Turicibacter | 0.44±0.47 | 1.40±0.92^#^ | 0.42±0.23^*^ | 1.98±1.11 |
|  | uncultured_bacterium_g__Lactobacillus | 0.42±0.39 | 1.39±1.53 | 0.87±0.53 | 1.26±1.28 |
|  | unclassified_g__Roseburia | 0.31±0.36 | 0.31±0.45 | 1.98±3.33 | 1.15±1.69 |
|  | unclassified_g__Eubacterium]_coprostanoligenes_group | 1.24±0.94 | 0.40±0.44 | 1.37±0.88^*^ | 0.71±0.36 |
|  | unclassified_g__norank_f__Ruminococcaceae | 1.20±0.62 | 0.62±0.50 | 0.77±0.57 | 0.61±0.92 |
|  | unclassified_g__Rikenellaceae_RC9_gut_group | 0.21±0.15 | 0.79±1.11 | 0.30±0.20 | 1.56±1.14 |
|  | uncultured_Bacteroidales_bacterium_g__norank_f__Muribaculaceae | 1.94±1.53 | 0.43±0.39^#^ | 0.06±0.07^*^ | 0.16±0.30 |
|  | uncultured_Allobaculum_sp._g__norank | 0.05±0.08 | 1.43±1.65^#^ | 0.14±0.14 | 0.90±1.44 |
|  | Blautia_glucerasea | 0.07±0.15 | 1.39±2.15 | 0.11±0.17 | 0.88±2.10 |
|  | unclassified_g__Veillonella | 0.02±0.01 | 1.12±2.46 | 0.09±0.12 | 1.29±3.26 |
|  | metagenome_g__Clostridium_sensu_stricto_1 | 0.66±0.86 | 1.09±0.76 | 0.52±0.42 | 0.01±0.01^*^ |
|  | Bacteroides_acidifaciens | 0.25±0.41 | 1.13±2.13 | 0.89±0.70 | 0.06±0.07 |
|  | uncultured_organism_g__Phascolarctobacterium | 0.00±0.00 | 1.30±2.04 | 0.00±0.01 | 0.45±0.95 |
|  | uncultured_bacterium_g__Ruminococcaceae_UCG-005 | 0.04±0.02 | 0.20±0.29 | 0.43±0.72 | 0.93±2.35 |
|  | Blautia_producta | 0.00±0.00 | 0.10±0.16 | 0.00±0.00 | 1.21±2.62 |
